# Aortic Arch Surgery: Progress, Perspectives and Future Challenges

**DOI:** 10.3390/jcdd13080386

**Published:** 2026-08-13

**Authors:** Jose Motta, Ravi De Silva, Jason Ali

**Affiliations:** Department of Cardiothoracic Surgery, Royal Papworth Hospital NHS Foundation Trust, Cambridge Biomedical Campus, Cambridge CB2 0AY, UK; ravidesilva@nhs.net (R.D.S.); jason.ali@nhs.net (J.A.)

The aortic arch remains one of the most challenging areas in cardiac surgery, requiring a unique combination of surgical accuracy, physiological insight, multidisciplinary collaboration, and intraoperative decision-making. While standardised protocols have been established for many cardiac procedures, aortic arch surgery still requires an individualised approach that balances immediate life-saving interventions with the goal of durable aortic reconstruction. This duality is perhaps most evident in the contrast between meticulously planned elective repair, in which multidisciplinary input—particularly radiologist-led preoperative imaging and device planning—shapes every stage of the pathway, and the compressed decision-making demanded by acute aortic syndromes such as type A dissection. Over the past two decades, advancements within surgical techniques, cerebral protection, prosthetic devices, and endovascular interventions have transformed patient management and outcomes [1]. Nevertheless, this is an evolving field, and ongoing questions about patient selection, neurological protection, optimal operative strategies, and long-term surveillance are driving this.

A major development in this field is the increasingly sophisticated understanding of aortic disease. Historically, thoracic aneurysms and acute dissections were considered distinct clinical entities with predictable management pathways. However, current evidence indicates that aortic disease is a heterogeneous spectrum influenced by complex interactions among vascular biology and haemodynamic and individual patient factors. Suslov and colleagues (Contribution 1) reveal that traditional associations, such as the link between atherosclerosis and dissection, are subtler than once believed. Their findings support the view that thoracic aortic degeneration cannot be explained solely by progressive atherosclerotic disease. Their data also indicate that patients with dissection tend to be younger than those without, underscoring age as a factor in individualised risk assessment. Likewise, Kletzer and colleagues (Contribution 2) remind us that classification systems should evolve alongside our understanding of disease, particularly in areas where anatomy and clinical behaviour do not conform to traditional paradigms. Patients with these conditions are classified as having dissections somewhere between conventional Stanford type A and type B, underscoring that anatomical classification alone is often insufficient to guide management. This heterogeneity extends beyond acquired disease: heritable and connective tissue aortopathies, including Marfan syndrome, Loeys–Dietz syndrome, and bicuspid aortic valve-associated aortopathy, add a further dimension to the disease spectrum, shaping surveillance intervals and surgical thresholds independently of the degenerative and dissection-related pathways discussed above.

The increasing recognition of disease complexity has directly shaped aortic surgical strategies. Modern aortic arch surgery emphasises selecting the most appropriate intervention for each patient, rather than routinely opting for the most extensive procedure. As highlighted by El-Andari and colleagues (Contribution 3), determining the optimal surgical strategy—whether hemiarch replacement, total arch replacement, hybrid repair, or a staged approach—requires a careful balance between patient-specific factors, anatomical complexity, surgical expertise, and institutional resources. The debate between conservative and extensive arch replacement has given way to a more individualised approach, in which the surgical strategy is tailored to the patient characteristics, disease complexity, and institutional expertise. This shift embraces a fundamental principle of contemporary aortic surgery: technical capability should expand treatment options but never supersede patient-centred decision-making. This complexity reinforces why arch surgery is increasingly concentrated within high-volume specialist centres; sustaining this expertise will depend on structured training pathways for the next generation of aortic surgeons and effective hub-and-spoke referral networks linking specialist and non-specialist centres.

The Frozen Elephant Trunk (FET) technique is one of the most significant contemporary innovations in aortic arch surgery [2]. Initially developed to enable staged management of extensive thoracic aortic disease, FET has become central to modern aortic arch reconstruction. The single-centre experience reported by Doll and colleagues (Contribution 4) demonstrates the constant refinement of this technique, which achieves acceptable early and mid-term outcomes even in complex patient populations. Their findings also highlight that although the prosthetics have advanced, significant challenges, like neurological injury, renal dysfunction, and the need for secondary interventions. The cost and availability of these advanced hybrid prostheses are a further consideration, and disparities in access to FET technology between high- and lower-resource healthcare systems warrant explicit attention as the field advances. Nevertheless, the FET has fundamentally reshaped aortic arch surgery by enabling comprehensive proximal repair while creating a durable platform for staged endovascular treatment when required.

Modern aortic surgical practice is now defined by the integration of open and endovascular surgical approaches. Endovascular reconstruction of the aortic arch has progressed from an experimental procedure to a viable therapeutic option for selected patients. As described by Guo and colleagues (Contribution 5), branched and fenestrated devices are expanding the scope of minimally invasive treatment. These techniques remain limited by factors like device complexity, stroke risk, and insufficient long-term data, but they could represent substantial progress towards individualised therapy for patients previously considered unsuitable for open surgery. In contrast to replacing open arch surgery, hybrid and endovascular procedures now complement classic surgical approaches, implying that collaboration will increase in the future of aortic surgery. Precision device selection and sizing, increasingly supported by advanced imaging reconstruction and emerging computational and machine-learning-based planning tools, will be central to translating these techniques safely into wider practice.

The success of aortic arch surgery relies on more than technical reconstruction. The procedure now constitutes one component of a comprehensive, multidisciplinary care pathway. Neurological protection remains an essential determinant of postoperative outcomes, with considerable improvements in both cerebral and spinal cord protection strategies [3] (Contribution 6). Two of the principal strategies underpinning this progress, moderate hypothermia and selective antegrade cerebral perfusion, continue to be refined, and neurocognitive recovery and quality of life after their use deserve greater attention alongside conventional measures of stroke and mortality. Furthermore, modern cannulation techniques have become essential for cerebral protection, reducing embolic risk, and maintaining distal organ perfusion (Contribution 7). Collectively, these developments signify a shift in focus: optimal outcomes are increasingly achieved by optimising the entire perioperative process rather than refining individual operative steps. Contemporary arch surgery therefore exemplifies the combination of expertise from surgeons, anaesthetists, perfusionists, intensivists, radiologists, and specialised nursing teams.

The systemic repercussions secondary to acute aortic disease are also reviewed throughout this Special Issue. Burysz and colleagues (Contribution 8) report that successful proximal aortic repair does not necessarily conclude the disease process. Instead, distal vascular remodelling, organ perfusion, and long-term surveillance continue to be essential aspects of patient care. Similarly, Demoulin and colleagues (Contribution 9) underscore that rare pathologies continue to challenge conventional treatment algorithms, stressing the importance of multidisciplinary decision-making in the absence of high-quality evidence.

Significant questions remain unresolved, despite remarkable progress in recent decades. Randomised evidence comparing open, hybrid, and fully endovascular approaches is limited, and optimal patient selection still relies primarily on institutional expertise and observational data. Artificial intelligence and machine-learning-based risk prediction tools may help refine this selection process, although their clinical validation remains at an early stage. The long-term durability of novel endovascular devices, strategies for neurological protection, surveillance protocols after hybrid repair, and methods to prevent distal aortic progression require further investigation. Enhanced collaboration through international registries, multicentre studies, and standardised reporting will be necessary to guarantee that forthcoming innovations are supported by robust evidence rather than technical enthusiasm alone. Established platforms such as the International Registry of Acute Aortic Dissection (IRAD) and the German Registry for Acute Aortic Dissection Type A (GERAADA) illustrate the value of this approach, and extending comparable collaborative infrastructure to hybrid and endovascular arch cohorts will be essential to ensure that progress in this field benefits patients equitably.

The evolution of aortic arch surgery represents the wider transformation of cardiac surgery. Success is now defined by the capacity to perform complex operations, as well as by the integration of surgical judgement, technological innovation, and multidisciplinary expertise to improve patient care. This Special Issue collectively demonstrates the specialty′s progress while drawing attention to ongoing challenges. As open, hybrid, and endovascular techniques become increasingly complementary, the future of aortic arch surgery will likely be shaped by the combination of evidence, innovation, and collaboration, rather than by any single operation or device. In this context, the future of aortic arch surgery appears equally dynamic as its recent past.

## Abbreviations

The following abbreviations are used in this manuscript:

FET	Frozen Elephant Trunk
IRAD	International Registry of Acute Aortic Dissection
GERAADA	German Registry for Acute Aortic Dissection Type A
